# First Case of Neuroblastoma‐Like Somatic‐Type Malignancy Arising in the Teratomatous Component of a Mixed Non‐Seminomatous Testicular Germ Cell Tumor in an Adult

**DOI:** 10.1002/ccr3.72216

**Published:** 2026-03-06

**Authors:** Karl Ziade, Jana Kotaich, Serge Assaf, Valerie Aftimos, Adib Ziade

**Affiliations:** ^1^ MEDICA Research Investigation Hadath Lebanon; ^2^ Faculty of Medical Sciences Saint Joseph University Achrafieh Beirut Lebanon; ^3^ Faculty of Medical Sciences Lebanese University Hadath Lebanon; ^4^ Notre Dame Des Secours University Hospital Center Byblos Lebanon; ^5^ School of Medicine and Medical Sciences Holy Spirit University of Kaslik Jounieh Lebanon; ^6^ Institut National de Pathologie Beirut Lebanon; ^7^ Chu Notre‐Dame Des Secours Jbeil Lebanon; ^8^ Hôpital Français du Levant Beirut Lebanon

**Keywords:** neoplasm, neuroblastoma, neuroectodermal tumor, non‐metastatic, testicle, testicular

## Abstract

Somatic malignant transformation (SMT) in germ cell tumors (GCTs) is a rare but clinically significant event. Among non‐seminomatous germ cell tumors (NSGCTs), teratomas can undergo malignant transformation, with neuroblastoma‐like differentiation being exceptionally rare. No standardized treatment protocols exist, and management is typically extrapolated from conventional approaches for GCTs and neuroblastoma. We report the first documented case of neuroblastoma arising within a testicular teratoma in a 25‐year‐old male presenting with a painless right testicular mass. Serum tumor markers showed elevated beta‐hCG, while AFP and LDH were normal. Imaging revealed no metastases. Radical orchiectomy was performed, and histopathology confirmed a mixed NSGCT with teratomatous and minor embryonal carcinoma components, with immunohistochemistry confirming neuroblastoma differentiation. The tumor was staged as pT1 (AJCC 8th edition). The patient completed four cycles of chemotherapy and remains in complete remission. Neuroblastoma arising within a testicular teratoma is an exceptionally rare occurrence, posing diagnostic and therapeutic challenges. Early diagnosis, histopathological evaluation, and multidisciplinary management are crucial for optimal outcomes. Further research is needed to refine treatment strategies for such rare malignancies.

## Key Clinical Message

This case highlights the first documented occurrence of neuroblastoma arising within a testicular teratoma, expanding the spectrum of SMT in GCTs. Given the rarity and chemoresistance of such malignancies, early recognition, comprehensive histopathological assessment, and multidisciplinary management are essential for optimizing patient outcomes. This case underscores the need for further research to establish standardized treatment protocols for SMT in GCTs.

**Abbreviations**
AFP: Alpha foetoprotein
FSH: follicle‐stimulating hormone
GCNIS: germ cell neoplasia in situ
GCT: germ cell tumors
HCG: human chorionic gonadotropin
LDH: lactate dehydrogenase
LH: luteinizing hormone
MSC: malignant somatic component
NB: neuroblastoma
SEER: surveillance epidemiology and end results

## 1 Introduction

Somatic malignant transformation (SMT) in germ cell tumors (GCTs) is a rare but clinically significant occurrence [1]. The morphological diversity of SMT remains poorly understood, and there is a lack of well‐established guidelines for its optimal therapeutic management [1]. Testicular GCTs are the most common malignancies in young adult males, with non‐seminomatous germ cell tumors (NSGCTs) comprising a significant subset [2]. Among NSGCTs, teratomas contain elements from all three germ layers and can, in rare instances, undergo malignant transformation, leading to the development of somatic‐type malignancies [2, 3]. The most frequently reported transformations include adenocarcinoma, sarcoma, and primitive neuroectodermal tumors, whereas neuroblastoma differentiation within a testicular teratoma is exceedingly rare [1, 3]. Neuroblastoma (NB) is a highly malignant, typically childhood cancer that arises from immature nerve cells (neuroblasts) of the sympathetic nervous system [2, 3]. While it is the most common extracranial solid tumor in children, its occurrence in adults is extremely rare, often presenting with a more aggressive course and poorer prognosis [2, 3]. In fact, adult‐onset NB is rare, with less than 0.3 cases per million per year, and an incidence of 0.2 cases per million for those aged 20–39 [4]. Adult survival rates are poor compared to children, with 3‐year and 5‐year survival rates of 45.9% and 36.3%, respectively [4].

GCTs are classified into three distinct types based on their developmental origin and association with germ cell neoplasia in situ (GCNIS): Type I GCTs include teratomas and yolk sac tumors that primarily occur in neonates and infants [3]. Type II GCTs originate from preexisting GCNIS and include NSGCTs, which predominantly affect adolescents and adults [3]. Type III GCTs consist solely of spermatocytic tumors (previously known as spermatocytic seminoma), which develop in older adults without a preceding GCNIS [3]. Over 90% of invasive GCTs belong to Type II tumors, as they originate from GCNIS [3, 5].

Malignant transformation of teratomas is hypothesized to result from genetic and molecular alterations in pluripotent germ cells, triggering their progression into aggressive neoplasms [1, 6]. While neuroblastoma is a well‐documented embryonal tumor in pediatric patients, its occurrence in adults, especially as a component of a mixed non‐seminomatous germ cell tumor, is exceptionally uncommon [1]. Given its rarity, there are no established treatment protocols, and management strategies are extrapolated from conventional approaches to both GCTs and neuroblastoma [2].

We report the first documented case of neuroblastoma arising in the teratomatous component of a mixed non‐seminomatous germ cell tumor of the testis in an adult male.

## 2 Case History/Examination

A 25‐year‐old male with no medical history presented with a palpable, stiff right testicular mass and a reducible right inguinal hernia. He was afebrile, with no chills or pain. Physical exam revealed a soft, non‐tender abdomen and no palpable lymph nodes. Blood tests, including urinalysis and chemistry, were normal, while hormonal tests showed elevated beta‐hCG (17.72 IU/mL) with normal alpha foetoprotein (AFP) and lactate dehydrogenase (LDH). Sperm analysis indicated asthenic terato‐oligospermia (5.8 million total count), and sperm preservation was advised. Sperm preservation was counseled and performed before any further treatment. Inguinoscrotal ultrasound with Doppler showed a right testis of average size 4.7 × 2.8 cm, with diffuse parenchymal microcalcification with a 40 × 36 mm hypoechoic parenchymal tumor, seeded with internal calcifications, with normal epididymis, no hydrocele and no varicoceles seen on Doppler during Valsalva. A thoraco‐abdominal and pelvic CT scan with contrast injection was performed for extension investigation showing no detectable metastasis nor lymph nodes.

## 3 Methods (Differential Diagnosis, Investigations and Treatment)

A radical orchiectomy was performed and sent to further pathological and immunohistochemistry studies [4]. The anatomopathological report shows a mixed germ cell tumor, non‐seminomatous, with a major teratomatous component including an undifferentiated component (90%) considered to be, on morphology, a somatic type malignancy and a minor embryonal carcinoma (10%).

**FIGURE 1:**
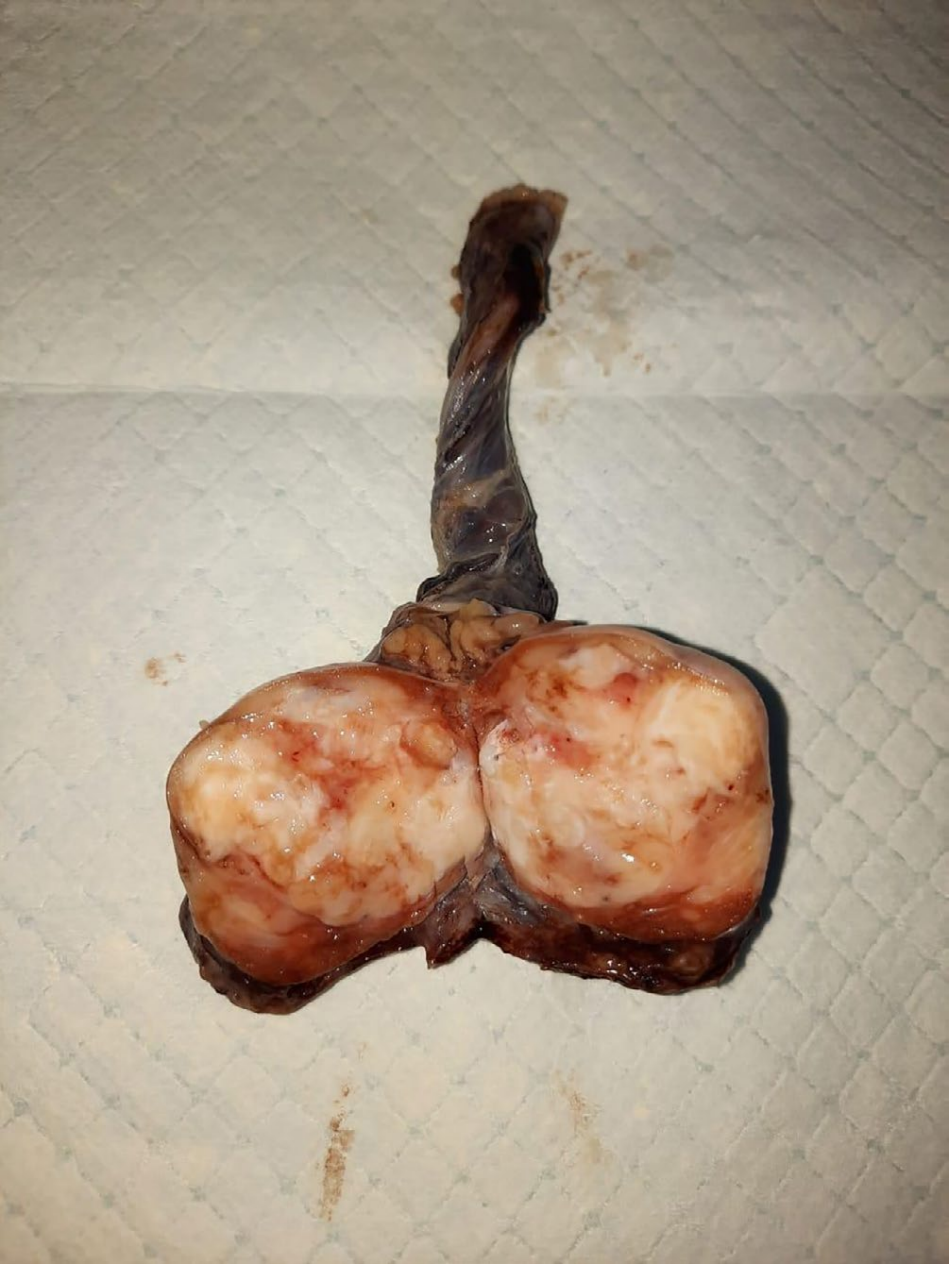
Gross appearance of the testicle: 4 × 3.5 × 3 cm heterogenous white tumor with a focal glistening appearance.

**FIGURE 2:**
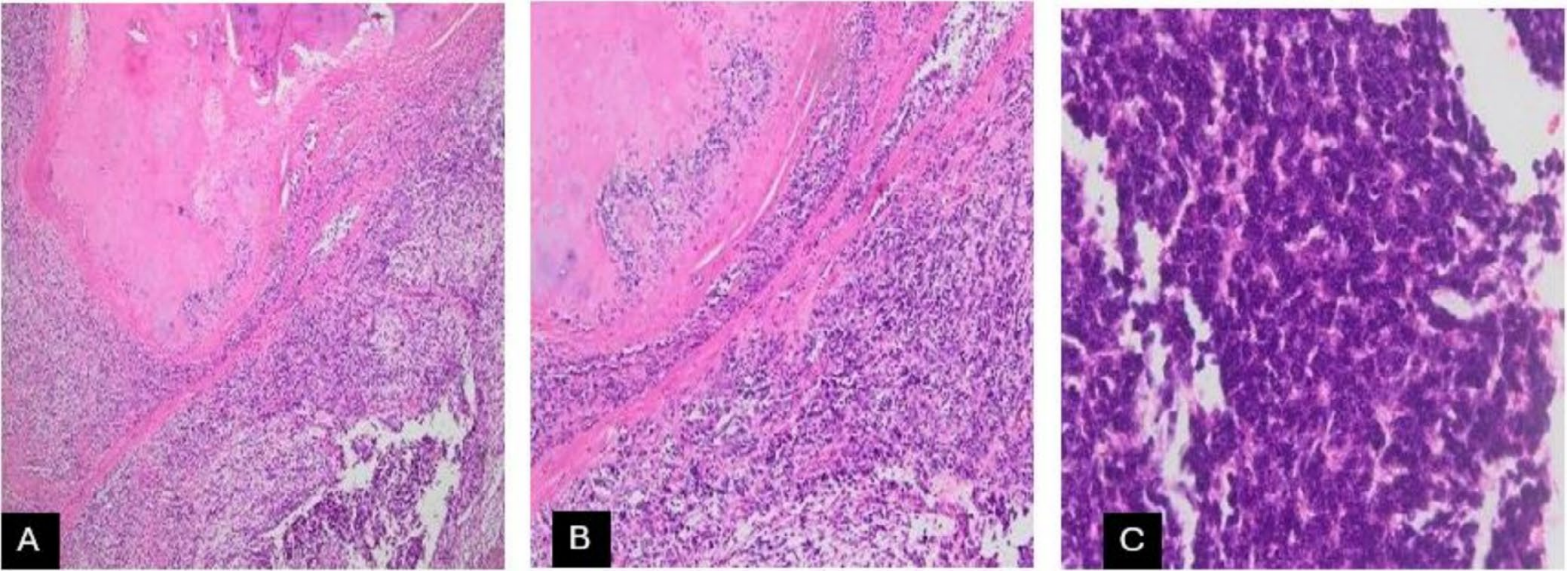
(A) The image shows the teratomatous component composed of cartilaginous lobules and the undifferentiated component (×4/H&E stain). (B) Higher magnification of the teratomatous and undifferentiated components of the tumor (×10/H&E). (C) The undifferentiated component shows small to medium‐sized basophilic cells with a scant cytoplasm and a salt and pepper chromatin. Some mitotic figures are seen (×40/H&E stain).

The teratomatous component was made up mainly of mature cartilage and some epithelial structures (Figure 1), along with the previously mentioned undifferentiated component. The latter was comprised of small to medium‐sized basophilic cells with scant cytoplasm and salt‐and‐pepper chromatin, arranged in fascicles and sheets (Figure 2).

**FIGURE 3:**
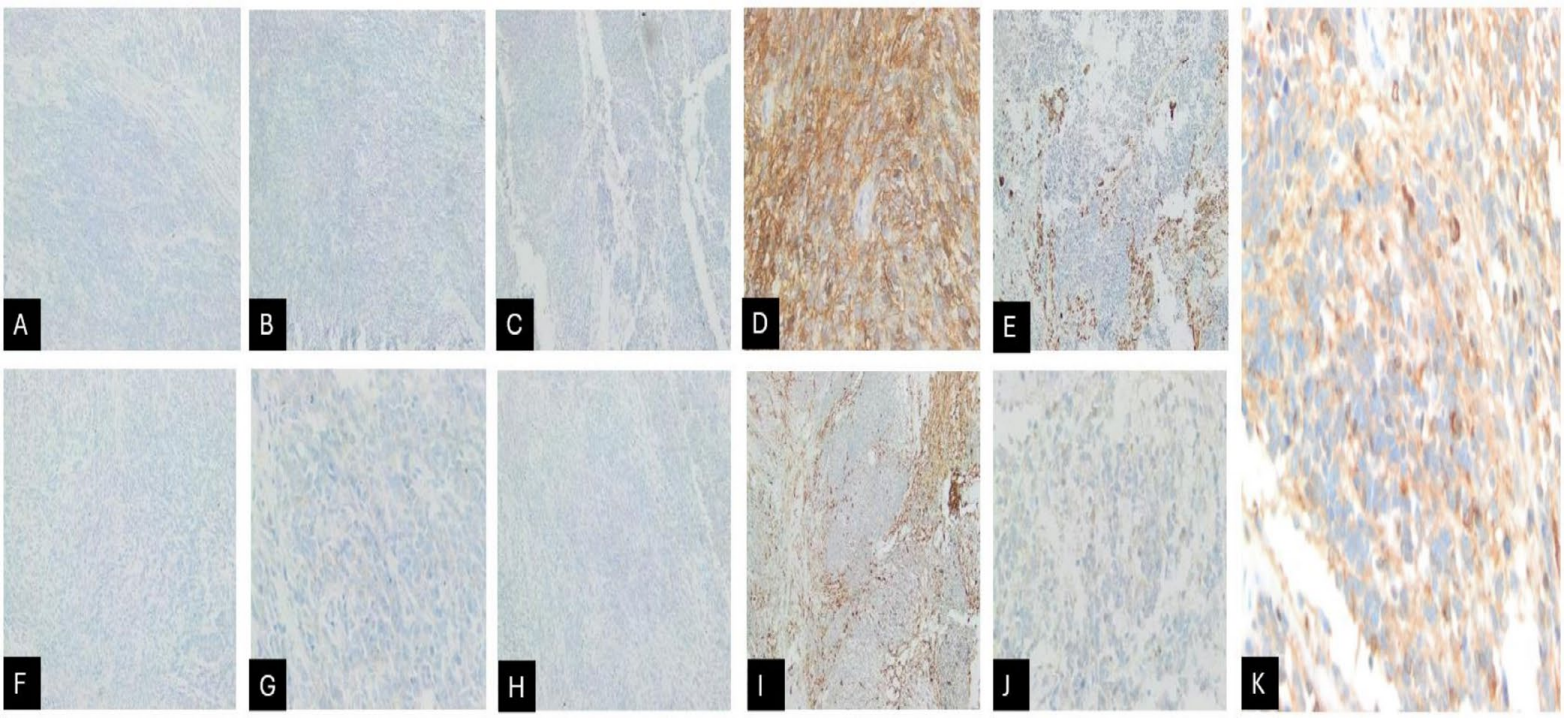
Immunostains on the undifferentiated component: (A) Beta HCG 10×. (B) Caldesmone 10×. (C) CD30 10×. (D) CD56 40×. (E) CK AE1/AE3 10×. (F) Desmin 10×. (G) Glypican 40×. (H) PLAP 10×. (I) PS100 10×. (J) Synaptophysin 40×. (K) NSE stain 40×.

Immunohistochemical study was performed to determine the nature of the undifferentiated tumor cell. The latter expressed in a diffuse manner CD56 and NSE, in a heterogeneous and weak manner, synaptophysin and in a focal manner PS100. Staining for CK AE1/AE3, CD30, PLAP, Beta‐HCG, Glypican, Desmin and Caldesmone are all negative in the undifferentiated component. CK AE1/AE3 is positive in the teratomatous component (Figure 3).

Based on the morphology and immunophenotype, the findings were considered most consistent with a somatic‐type malignancy showing neuroblastoma‐like differentiation arising within a testicular teratoma. The TNM staging, as per the 8th AJCC edition, is pT1 [5], with a tumor measuring 4 cm in largest diameter, with invasion of the rete testis but no invasion of the epididymis, the tunica albuginea, or tunica vaginalis. No vascular invasion was detected. The surgical margin is clear.

## 4 Conclusion and Results (Outcome and Follow‐Up)

Our patient has completed four cycles of chemotherapy and remains in complete remission. A recent abdominal CT scan showed no residual disease, and ongoing monthly surveillance includes tumor markers, CBC, and liver/kidney function tests.

## 5 Discussion

Various somatic‐type malignancies have been described in GCTs, including sarcomas, carcinomas, and primitive neuroectodermal tumors [7]. We report the first documented case worldwide of an undifferentiated neuroblastoma arising within a testicular teratoma in an adult.

Classifying testicular tumors is challenging due to their rarity and structural overlap with other entities [8]. In this context, definitive diagnosis relies on careful morphologic assessment supported by immunohistochemistry to distinguish SMT from other primary testicular neoplasms [3].

In GCT with malignant somatic components (MSC), like neuroblastoma in a testicular teratoma, MSCs are less responsive to chemotherapy, making radical surgery crucial for better outcomes. Early‐stage diagnosis and complete surgical resection are key prognostic factors, especially in advanced cases with otherwise poor prognosis [7].

The mechanisms underlying secondary transformation in teratomas remain a subject of ongoing investigation. It has been suggested that the emergence of rare tissue types within germ cell tumors, including derivatives of the neural crest, could result from neometaplasia driven by totipotent stem cells [9]. Secondary malignant transformations occur in about 2% of mature teratomas and may give rise to various malignancies, such as squamous cell carcinoma, papillary thyroid carcinoma, and carcinoid tumors [10]. In cases of mature teratomas, prolonged persistence of the lesion is believed to elevate the risk of secondary malignancy, as observed in de novo squamous cell carcinoma [11]. Molecular analyses have identified p53 mutations as a potential driver of tumorigenesis in these secondary malignancies [12]. In our case, the testicular mass displayed a well‐defined structure with both immature neuroepithelium and mature elements from all three germ layers, supporting the likelihood that the neuroblastoma arose through secondary transformation of the immature neuroepithelium within the teratoma. Another possible explanation is a collision tumor phenomenon, where two distinct neoplasms coexist within the same lesion. While rare in testicular tumors, this phenomenon has been described in mature teratomas associated with epithelial malignancies [13].

In discussing testicular neuroblastoma within a teratoma, it is useful to compare it with similar cases, like primary ovarian neuroblastoma in a teratoma. Both highlight diagnostic challenges and the role of immunohistochemistry in confirming neuroectodermal origins. Prognosis is variable, often more favorable in younger patients. The absence of poor prognostic markers, such as N‐myc amplification, may suggest a more favorable outlook with careful monitoring. This comparison emphasizes the importance of recognizing and managing neuroblastomas within teratomas across gonadal sites, adding valuable insights to the limited literature on these rare tumors [14, 15].

The diagnosis of neuroblastoma in this case warrants cautious interpretation. While the undifferentiated component demonstrated morphologic features compatible with neuroblastic differentiation and expressed neuronal markers including synaptophysin, CD56, and NSE, definitive confirmation of neuroblastoma ideally requires demonstration of neural crest lineage using PHOX2B immunohistochemistry. PHOX2B staining could not be performed due to unavailability in our country, and its absence represents an important limitation. Similarly, auxiliary diagnostic investigations such as urinary HVA/VMA levels, MIBG scintigraphy, and MYCN amplification analysis were not available, further limiting definitive subclassification. Nevertheless, the overall morphologic, immunophenotypic, and clinical context supports the interpretation of a neuroblastoma‐like somatic‐type malignancy arising within a teratoma.

Should PHOX2B immunohistochemistry become available through external testing, it would provide valuable confirmatory evidence and further refine the diagnostic classification of this rare entity.

This case enhances our understanding of rare testicular tumors and highlights the importance of thorough histopathological assessment and multidisciplinary management for optimal outcomes.

Further research is needed to better understand the pathogenesis and optimal management of these rare tumors.

## Author Contributions

**Karl Ziade:** conceptualization, investigation, methodology, visualization, writing – original draft, writing – review and editing. **Jana Kotaich:** conceptualization, investigation, methodology, project administration, visualization, writing – original draft, writing – review and editing. **Serge Assaf:** investigation, methodology, resources, writing – original draft. **Valerie Aftimos:** investigation, methodology, project administration, supervision, writing – original draft, writing – review and editing. **Adib Ziade:** data curation, investigation, resources, supervision, visualization, writing – review and editing.

## Funding

The authors have nothing to report.

## Ethics Statement

The authors have nothing to report.

## Consent

Written informed consent was obtained from the patient to publish this report in accordance with the journal's patient consent policy.

## Conflicts of Interest

The authors declare no conflicts of interest.

## Data Availability

No datasets were generated or analyzed for this case report.
